# Identification of maladaptive behavioural patterns in response to extreme weather events

**DOI:** 10.1038/s41598-024-60632-3

**Published:** 2024-05-08

**Authors:** Luisa Eusse-Villa, Carolina Bonardi Pellizzari, Cristiano Franceschinis, Mara Thiene, Marco Borga, Anna Scolobig

**Affiliations:** 1 Land, Environment, Agriculture and Forestry Department, University of Padova, Legnaro, Italy; 2 Institute for Environmental Sciences, University of Geneva, Geneva, Switzerland; 3 Equity and Justice Group, International Institute for Applied Systems Analysis, Laxenburg, Austria

**Keywords:** Environmental social sciences, Natural hazards

## Abstract

Human behaviour has gained recognition as a critical factor in addressing climate change and its impacts. With extreme weather events posing risks to vulnerable communities, understanding cognitive processes driving behaviours becomes essential for effective risk communication. This study focuses on the 2018 “Vaia” storm, which brought unprecedented precipitation and wind velocity to the mountainous regions of North-eastern Italy. Drawing upon the Protection Motivation Theory (PMT) framework, we employ probabilistic models to identify distinct groups with similar behavioural profiles. By administering a web-based survey to 1500 residents affected by the event, we find that threat appraisal is more influential in shaping protective behaviours than coping appraisal. Our findings indicate that by enhancing coping appraisals and discouraging non-protective measures, we can actively mitigate maladaptive responses and promote the adoption of effective adaptation strategies.

## Introduction

Human behaviour has been historically neglected in climate science, but not anymore. It is now widely recognized that human behaviour not only contributes to climate change but also plays a crucial role in addressing and mitigating its impacts. The latest Intergovernmental Panel on Climate Change (IPCC) report^1^ emphasizes the need for a synergistic approach to climate change mitigation and adaptation while highlighting the importance of avoiding maladaptive responses that can further exacerbate the effects on human-environment systems. The intensification and increased frequency of extreme events, such as storms, floods, landslides, droughts, and fires, have profoundly and notably impacted both nature and human life^1,2^. These events result in escalating loss of life, infrastructure damage, food insecurity, and population displacement. Recent examples, like the devastating flood in Pakistan in 2022 and the heatwaves and droughts in Europe, underscore the urgency of addressing the role of human behaviour in mitigating climate change impacts as numerous studies have established a strong connection between anthropogenic activities, the emission of greenhouse gases, climate change and its impacts on hydrological cycles, manifesting in phenomena like droughts and floods^3–7^. Understanding the cognitive processes that drive behaviours related to climate change, including responses to extreme events, is vital for effective risk communication and developing strategies to reduce vulnerability of residents^8^ and maladaptation.

Maladaptation refers to “*inappropriate responses to climate change which create long-term lock-in of vulnerability, exposure and risks that are difficult and costly to change*”^1^. This issue is often rooted in insufficient knowledge, lack of resources and short-term focused governance approaches^1,9^. Maladaptation can be classified into structural, institutional, and behavioural^10^. An illustrative example is the construction of a seawall in Venice intended to mitigate sea-level rise, which unintentionally disrupted the local ecosystem by altering water flow and sediment deposition dynamics^11^. Moreover, certain socio-economic groups face greater vulnerability and exposure, leading to increased flood impacts if adaptation measures are not equitably distributed^12^. Additionally, prevailing approaches to flood risk assessment often overlook critical interactions between humans and flood systems^13^. To mitigate maladaptation, inclusive governance, human and economic resources availability and increased knowledge are crucial, emphasizing the significance of public engagement in disaster risk management through adaptation and mitigation measures, particularly at the individual level^14^. For instance, a study focused on the river Rhine and its flood plains estimated that monetary flood damages in flood-prone areas could be reduced by 80% through individual protection behaviours such as implementing private mitigation measures like floodwalls or floodgates^15^. Various factors, including risk perception and previous experiences with climate change-induced events, influence these protection behaviours^16^.

Efforts to enhance risk awareness and promote individual adaptation behaviours have gained attention from decision-makers and researchers^17–19^. The Protection Motivation Theory (PMT) has been employed within the flood-risk context to understand individual responses^20,21^. The PMT encompasses the process of how people receive information about a hazardous event, evaluate the risks and the effectiveness of existing measures, and subsequently develop adaptive or maladaptive behavioural responses^20^. Originally proposed in 1975^20^ and revised in 1983^21^, the PMT was initially used in the domain of health issues and disease prevention but has since found application in various contexts, including disasters such as landslides, hurricanes, and wildfires^14,19,22–25^. When applied to the analysis of individuals’ protection behaviour in the context of flood events, numerous studies have highlighted the significant role played by the theory’s components in explaining individual adaptation behaviour. Thus, the applications of the PMT provide valuable insights for decision-makers, enabling them to tailor communication efforts towards promoting individual protection behaviour^25–33^.

The PMT involves two cognitive processes that influence the adoption of protective behaviours: threat appraisal and coping appraisal. These processes are influenced by various factors that positively or negatively affect the likelihood of engaging in a response^21^. For instance, coping appraisal is positively influenced by factors such as response efficacy and self-efficacy, while it is negatively affected by the perceived costs of adopting a recommended response. According to the PMT, individuals are more likely to engage in protective behaviours when their threat appraisal is high (indicating a perception of the severity of the threat and vulnerability) and their coping appraisal is high (indicating a belief in the effectiveness and feasibility of protective behaviours). These individuals are classified as “problem-focused” within the PMT framework. Conversely, individuals who perceive a high threat but have a low coping appraisal, i.e., they do not perceive available protective measures as effective, easy, or affordable, are classified as “maladaptive” within the theory. Additionally, there are two other combinations of high and low appraisals in threat and coping that lead to the strategies of “no action” (low threat and low coping appraisals) and “just-to-be-sure” (low threat and high coping appraisals)^34,35^.

The threat and coping appraisals, and ultimately the individual’s protection behaviour, are significantly influenced by sources of information, which constitutes another important component of the PMT theory. These sources include an individual’s previous experiences with similar events, communication from various media channels (such as the internet, television, radio, and social media), interpersonal relationships with neighbours, family, and the community, as well as observations of others’ behaviours^21–24^. Previous experiences have a positive impact on threat appraisal, as individuals who have encountered similar events tend to perceive higher levels of threat and exhibit greater risk awareness. Conversely, individuals with limited or no prior experience with such events may have lower threat appraisals and reduced risk awareness^17,26,36^.

This study aims to investigate the factors influencing individuals’ adaptive behaviour towards hazardous events using empirical data within the theoretical framework of the PMT. Moreover, it explores the possibility of categorizing respondents into distinct groups with similar profiles regarding their protective behaviour. The empirical evidence utilized in this research is derived from the Vaia storm, an extreme event that occurred in Northern Italy in 2018. This event stands out as one of the highest recorded instances of cumulative precipitation and wind velocity in Italy^37^. The Vaia storm was characterized by powerful winds and excessive precipitation, resulting in windthrow, floods, and landslides that significantly impacted the local ecosystem, economy, and society^38^. The damage caused by the storm encompassed approximately 41,000 hectares of forest and led to the loss of approximately 8.5 million cubic meters of wood^39^.

To gain insights into protective behaviours associated with the Vaia storm, a web-based survey was conducted among approximately 1500 inhabitants of the Veneto and Trentino Alto Adige regions, which were severely affected by the event. The survey, implemented by a marketing agency in 2019, aimed to document behavioural responses related to the Vaia storm quantitatively. It covered a range of topics, including changes in daily routines, the effectiveness of warning communication, personal damages suffered, pre-and post-event protective measures taken, as well as attitudinal and psychological traits, specifically related to the components of the PMT. The survey focused on three main behavioural aspects of interest: (i) pre-storm protective measures, (ii) post-storm protective measures, and (iii) changes in regular activities. By employing the PMT, we aimed to identify the key components influencing respondents’ cognitive processes and the significant sources of information for them.

Furthermore, to explore the existence of distinct groups with similar protective behaviours in response to the catastrophic event, we employed probabilistic models known as Latent Class Clusters (LCC). This approach offers several advantages for our study. Not only does it enable the grouping of individuals based on their protective behaviours, but it also allows for population-wide inferences using statistical models. Additionally, statistical criteria can be used to determine the optimal number of groups while assessing the statistical significance of the estimated parameters^40^. The application of LCC models provides valuable insights into understanding different behavioural patterns and aids in directing targeted efforts toward specific groups^40^.

## Methods

### Survey implementation

To explore the factors influencing individuals’ choices to adopt risk adaptation strategies, we conducted a web-based survey among approximately 1500 residents of the Veneto and Trentino Alto Adige regions. The survey, conducted in 2019, aimed to capture behavioural responses associated with the Vaia storm event quantitatively. The questionnaire covered various aspects, including whether respondents altered their daily routines during the storm and the reasons behind those changes, the information they received before and during the event and their reactions to it, the extent of damage they experienced, changes in risk awareness following the event, personal protective measures taken before and after the event, as well as their attitudinal and psychological characteristics, particularly focusing on the principles of the PMT^20,21^. In accordance with established guidelines and regulations, all methods outlined in this study were conducted to adhere to ethical standards. The need for ethics approval was waived, as granted by the Institutional Review Board with the assessment number Prot. n. 0000808 of 07/03/24. The President of the Ethical Committee for the Research at the Department of Land, Environment, Agriculture, and Forestry from the University of Padova provided this approval. The data for this study was collected by a third-party marketing agency, which obtained informed consent from all respondents in compliance with ethical standards. Only individuals aged 18 and above were included in the study. Prior to their participation, all subjects were provided with comprehensive information about the study, and informed consent was obtained to confirm their voluntary and knowledgeable participation in the research.

### Behaviour of interest

The behaviours considered in this research involved structural and emergency preparedness measures, such as building drainage channels in the property or having a first aid kit. Those measures were grouped depending on the moment they were taken: (i) before and (ii) after the storm. The third behaviour category was related to whether the respondents changed activities they usually carry out on a regular day, such as grocery shopping. The timeline for these measures aligns with the questionnaire’s distinction between actions implemented before the storm (prior to October 29th, 2018) and those undertaken in the months following the storm.

For each of the three behaviour categories, a series of questions were asked (Supplementary Table 1), and if the respondent carried out at least one of the measures presented in the questionnaire for that category, she/he would receive a score of 1, as opposed to those who didn’t adopt any measure, who received a score of zero.

### Factors that influence individuals’ adaptive behaviour

In order to identify the factors that influence individuals’ adaptive behaviour toward the extreme event, an OLS regression analysis was carried out. We used as behaviour of interest the three dummy variables previously presented in the item above. As explanatory variables, we used the respondent’s self-reported ratings—on a scale of 1 (disagree) to 5 (completely agree)—of all the components of the PMT, in addition to sociodemographic characteristics and sources of information (Supplementary Table 2).

The effects of the explanatory variables on protection behaviours are estimated using OLS regression models using the statistical software R. Separate linear regressions were calculated considering each of the three dependent variables related to protection behaviour. We only included explanatory variables with Pearson correlation coefficients below 0.70 in the regressions. We found only one variable, namely “looked for more information,” showing correlations above 0.70; therefore, we removed it from the analysis. All the statistically significant variables (*p*-value < 0.1) were included in the results of each regression.

### Profiles of adaptive behaviour

To examine whether there is a segregation of respondents in different groups exhibiting similar profiles in terms of protection behaviour towards the catastrophic event under study, probabilistic models^40,41^ were estimated. The probability of belonging to different behavioural groups was explained by respondents’ self-reported attitudinal statements regarding the components of the PMT, particularly threat and coping appraisals. In addition, individuals’ characteristics, such as socio-demographics and other covariates capturing environmental and intrapersonal sources of information, were used to explain the segregation of groups of households (Supplementary Table 2). To carry out the LCC we used the R package poLCA^42^. The literature suggests using information criteria (e.g., Akaike Information Criterion, Bayesian Information Criterion) to select the optimal number of latent classes, where the optimal number of classes is given by the best-performing model, which minimises the score of the information criteria^43,44^. We estimated a range of models from 2 to 7 classes using the abovementioned criteria to define the optimal number of classes. Nevertheless, given that the information criteria values were minimized each time a new class was added, we could not provide evidence supporting a specific number of classes. Therefore, based on the PMT prediction groups, we decided that the four-class model was the most appropriate for our study.

## Results

**Figure 1:**
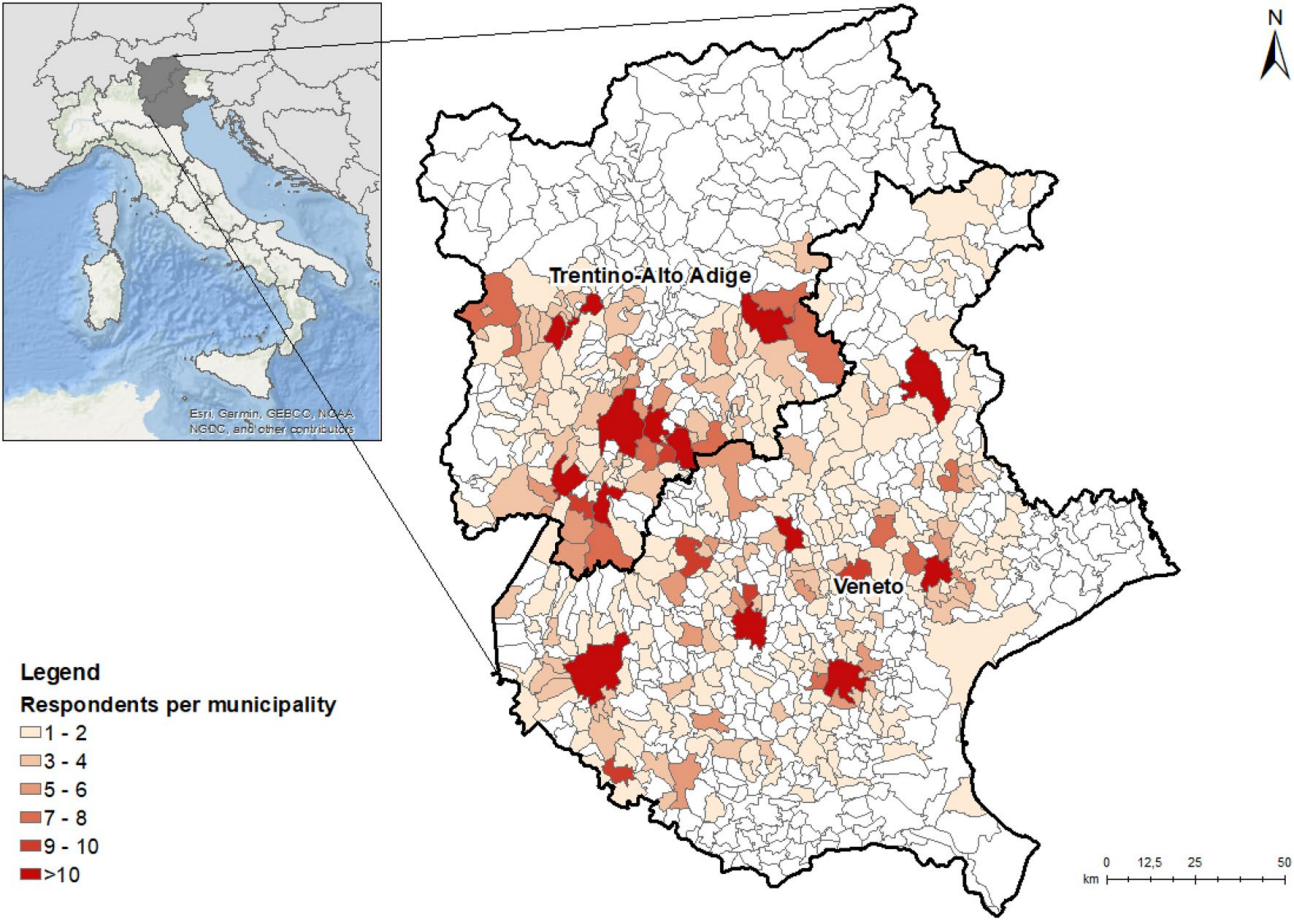
Geographic distribution of respondents. Respondents are located inside municipalities of two Italian regions (Trentino-Alto Adige and Veneto). Some municipalities have more respondents than others (indicated in red). The map was generated using ArcGIS (version 10.8.2, Esri Inc., Redlands, CA, USA^45^). For detailed information on the software, including the version mentioned, please refer to https://www.esri.com/en-us/arcgis/products/arcgis-desktop/resources.

A total of 1388 respondents participated in this study, with 703 (50.6%) identifying as female and 685 (49.4%) as male. The respondents represented a wide age range, spanning from 19 to 80 years old, with an average age of 47. Regarding education, 413 (29.8%) respondents held a university diploma. Overall, the sample used in this study can be considered quite representative of the targeted regions’ population, capturing various demographic characteristics. Regarding property ownership, most respondents owned the properties affected by the Vaia storm, while 17.1% were tenants (Table 1). Geographically, 840 (60.5%) respondents were located in the Veneto Region on the day of the storm, while 548 (39.5%) were in Trentino Alto Adige. The respondents were distributed across 331 municipalities in six provinces within these regions (Fig. 1).

**Table 1:** Descriptive statistics for the sample.

Variable	Category	Sample distribution (%)
Region	Trentino Alto Adige	39.5
	Veneto	60.5
Gender	Female	50.6
	Male	49.4
Age (years)	< 19	3.2
	20–39	37.3
	40–59	37.2
	> 60	22.3
Education	University degree	29.8
	High school	43.9
	Middle school	14.7
	Elementary school	1.7
	Professional qualification	9.9
Ownership	Owner	82.9
	Tenant	17.1
Income (€/year)	< 15,000	15.9
	15,001–30,000	35.2
	30,001–45,000	22.0
	45,001–60,000	8.0
	> 60,000	2.8
	I’d rather not answer	16.1

### Behaviours of interest

A significant portion of the respondents, approximately 90% (n = 1242), reported implementing at least one protective measure usually recommended for storm events before the day of the Vaia storm. These measures included actions such as securing objects at risk, pruning trees, or constructing temporary barriers. However, the number of respondents who implemented protective measures in the months that followed the storm decreased to 31% (n = 426). Additionally, a substantial number of respondents, 46% (n = 634), reported renouncing at least one routine or planned activity on the day of the storm (Supplementary Fig. 1.).

### Factors influencing protection behaviour

The regression analyses revealed that the PMT constructs significantly influenced the adoption of protective behaviour (Table 2). Threat appraisal exhibited a stronger influence than coping appraisal, particularly for protective measures implemented after the storm. Fear was found to be statistically significant (*p*-value < 0.01) and positively correlated with all three dependent variables, indicating that individuals with higher levels of fear tended to engage more in protective behaviour, especially after the storm. Perceived vulnerability and perceived severity, the other components of threat appraisal, also significantly influenced behaviour. Perceived vulnerability was statistically significant for protective measures after the storm and for renouncing activities, while perceived severity was significant for all three dependent variables.

**Table 2:** OLS regression results.

	Protective measures		Renounced activities
	Before Vaia	After Vaia	
R^2^	0.0946	0.1082	0.1596
Intercept	0.8922***	0.2019*	0.5796***
Threat appraisal			
Perceived vulnerability	ns	− 0.0560**	− 0.0643***
Perceived severity	0.0230	0.0376*	0.0759***
Fear	0.0244**	0.0892***	0.0542***
Coping appraisal			
Response efficacy	ns	− 0.0327	− 0.0336
Self-efficacy	ns	ns	ns
Response costs	ns	ns	ns
Sociodemographics			
Gender	ns	− 0.0722**	ns
Age	− 0.0042***	− 0.0022**	− 0.0051***
Education	ns	ns	− 0.0524
Income	ns	0.0633*	ns
Ownership	ns	ns	ns
Sources of Information			
Previous experience	0.0439*	ns	0.0488
Volunteer activities	ns	0.1707***	0.1882***
Information	ns	ns	ns
Personality traits	ns	ns	ns
Source of Information	ns	ns	ns
Risk zone	ns	0.1019***	ns

*ns* not significant. Signif. Codes: ‘***’ 0.001 (99.9%) ‘**’ 0.01 (99%) ‘*’ 0.05 (95%) ‘.’ (90%).

In terms of coping appraisal, the results indicated that response costs and self-efficacy did not significantly influence the implementation of protective measures. Response efficacy was the only component that significantly affected protective behaviour, showing a negative coefficient. The results regarding the influence of information sources were mixed. Only past experience was found to significantly influence the adoption of protective measures before the storm. Interestingly, receiving information about the event did not significantly influence protective behaviour, but engaging in volunteer activities was positively and significantly associated with protective measures and renouncing activities. Living in a risk zone significantly influenced the adoption of protective measures after the storm. Among the socio-economic variables, age emerged as an important factor explaining all the dependent variables, with a negative coefficient indicating that older individuals took fewer protective measures.

### Types of adaptation strategies in the context of climate change

**Figure 2:**
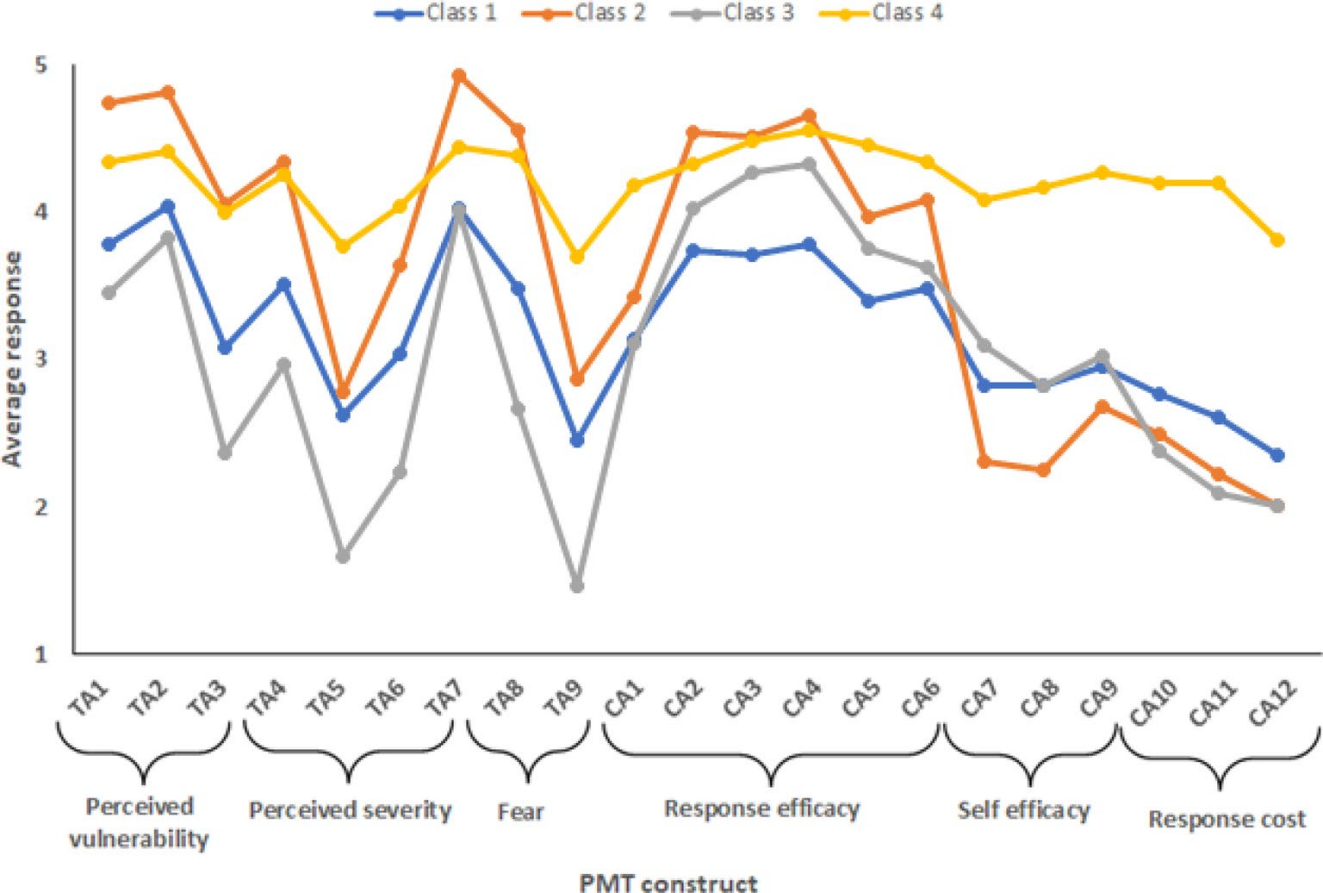
Average estimated response levels by question and class weighted by estimated probabilities. The threat appraisal component is denoted by the variables TA (1–9), while the coping appraisal is by the variables CA (1–12) (refer to Supplementary Table 1 for details on the PMT constructs).

The results from the LCC models indicate that the respondents can be categorized into four distinct groups or classes (Supplementary Table 3). The largest class, Class 1, represents 37% of the respondents, followed by Class 2 with 33%, Class 3 with 22%, and Class 4 with 8.5%. Figure 2 presents the average estimated response levels by question and class, weighted by estimated probabilities for each component of the PMT theory.

**Figure 3:**
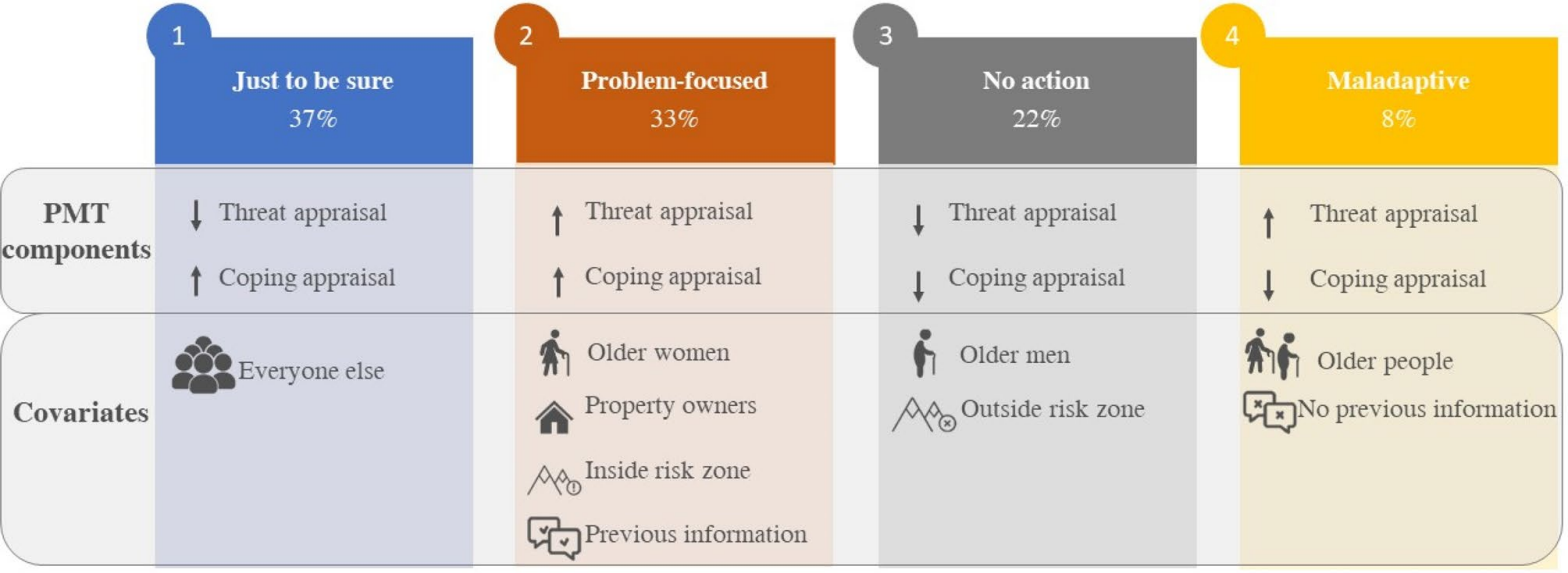
Groups classification. Details of each group based on PMT components and additional information based on sociodemographic and previous information.

In addition to the PMT questions, socio-economic data and sources of information provide further insights into group membership (Table 3). Figure 3 highlights the groups’ details. A significant subset of respondents (33% of the sample) is characterized by high threat appraisals and high coping appraisals, representing a “problem-focused” coping strategy (Class 2). These individuals are likely to be older women who own the affected property and have previous experiences with similar events. They also reside in a risk zone area and sought additional information about the storm before it occurred.

**Table 3:** Class membership function for the LCC model.

Classes	Just to be sure		Problem focused		No action		Maladaptive	
Class size	37%		33%		22%		8%	
Covariates	Coeff	*p*-value	Coeff	*p*-value	Coeff	*p*-value	Coeff	*p*-value
(Intercept)	–	–	− 272.546	< 0.001	− 189.700	< 0.001	− 287.736	< 0.001
Age	–	–	*0.034*	< 0.001	*0.033*	< 0.001	*0.024*	0.037
Woman	–	–	*0.488*	0.004	− *0.405*	0.042	− 0.016	0.965
Higher education level	–	–	0.012	0.947	0.158	0.464	0.284	0.447
Higher income	–	–	− 0.192	0.259	− 0.171	0.403	− 0.553	0.120
Owner	–	–	*0.509*	0.023	− 0.043	0.858	0.336	0.372
Previous flood experience	–	–	0.344	0.069	0.112	0.619	− 0.200	0.683
Participated in volunteer activities	–	–	− 0.045	0.899	0.681	0.064	139.497	0.013
Received information about the event	–	–	− 0.591	0.225	0.456	0.374	− 114.539	0.370
Look for additional information about the event	–	–	*0.268*	0.028	*− *0.045	0.745	0.458	0.117
Source of information	–	–	0.135	0.656	*− *0.004	0.990	*− 147.885*	0.025
Lives in a risk zone	–	–	*0.427*	0.022	*− 0.519*	0.048	0.201	0.609

Coefficients rendered in italics indicate statistical significance.

Moreover, 22% of the respondents show low scores in both threat and coping appraisals, indicating that they do not feel affected by the problem (Class 3). This group, referred to as the “no action” class in the PMT theory, primarily comprises individuals, most likely older men, who do not live in a risk zone area. A small portion of the sample (8%) is classified as the “maladaptive” coping strategy (Class 4), characterized by high threat appraisal and low coping appraisal. The high response costs associated with protective measures mainly influence the low coping appraisal. Individuals within this class, probably older men, did not actively seek information about the event before it happened. Lastly, a majority of respondents (37%) align with the “just to be sure” class (Class 1) per PMT theory. They exhibit high coping appraisals but low threat appraisals, indicating that they do not perceive the threat as severe but believe that the existing measures are effective. Class 1 encompasses all other respondents in our sample.

To assess the consistency between the class profiles identified and the respondents’ behaviour regarding renouncing activities, we examined the correspondence between classes and actual behaviour (Table 4). The results indicate that most individuals who renounced activities belonged to the “problem-focused” class, which aligns with their high threat and coping appraisals. On the other hand, the respondents who renounced fewer activities were more likely to belong to the “maladaptive” class, characterized by high threat appraisal but low coping appraisal. These findings suggest that there is a correspondence between the identified class profiles and the behavioural patterns observed in terms of renouncing activities. The “problem-focused” class, with higher threat and coping appraisals, appears to be more proactive in taking protective actions, including renouncing activities. In contrast, the “maladaptive” class, with high threat appraisal but low coping appraisal, tend to exhibit less proactive behaviour in terms of renouncing activities.

**Table 4:** Engagement in protection activities per class.

Renounced activity (total of respondents)	Just to be sure	Problem focused	No action	Maladaptive
	(n = 508)	(n = 461)	(n = 303)	(n = 116)
Staying home (153)	31.4%	35.9%	18.3%	14.4%
Going to work (177)	30.5%	37.3%	16.9%	15.3%
Going to study (school/university) (79)	53.2%	26.6%	12.7%	7.6%
Going grocery shopping (239)	34.7%	40.6%	16.3%	8.4%
Going to accompany/pick up children from school or other activities (131)	32.1%	38.2%	16.8%	13.0%
Going to visit relatives and/or friends (174)	32.2%	42.0%	16.7%	9.2%
Playing sports (170)	37.6%	35.9%	17.6%	8.8%
Practising outdoor hobbies (175)	25.7%	49.7%	19.4%	5.1%
Doing volunteering activities (72)	34.7%	34.7%	15.3%	15.3%

A percentage of the respondents that renounced a listed activity is shown by each of the classes.

## Discussion

In this study, we aimed to examine the factors that influence individuals’ adaptive behaviours in response to the Vaia storm before, during, and after the event. Our regression analysis evidenced the influence of the Protection Motivation Theory’s (PMT) constructs on respondents’ protective behaviour. In terms of threat appraisal, the observed effect of perceived vulnerability may be attributed to a lower perception of risk among individuals who had already taken protective measures before the Vaia storm. This finding aligns with previous studies that have reported similar results^28,31^. The positive coefficient for perceived severity, indicating that higher perceived severity was associated with increased protective behaviour, is consistent with recent research conducted in other countries^46^. This suggests that individuals who perceive the storm’s potential consequences as more severe are more likely to engage in protective behaviours.

Regarding coping appraisal, our results indicated that response costs and self-efficacy did not significantly impact the implementation of protection measures. The lack of significance for response costs aligns with findings from previous studies^28,32,47^. However, our finding differs from some previous research that reported significant and positive effects for self-efficacy^32,47^ or negative effects^28^. Interestingly, the only component of coping appraisal that significantly influenced protective behaviour in our study was response efficacy, which exhibited a negative effect. This finding deviates from the literature, where response efficacy has typically been found to positively influence protective behaviour^32,47–49^.

The results about the influence of different sources of information on protective measures exhibit mixed findings. Interestingly, in our study, the only variable that significantly influenced the adoption of protection measures before the Vaia storm was previous experience. This finding aligns with previous research^31,47,50^ that has demonstrated the impact of first-hand experience on adopting mitigating measures. For example, a study conducted in Italy found that individuals who had experienced a flood event were more likely to adopt mitigating measures in their study area^17^.

Regarding the socio-economic variables, the negative effect observed for age could potentially be explained by the “time effect.” This effect suggests that individuals who have resided in the area for longer and have already implemented various measures over time may be less inclined to undertake additional ones. This implies that older individuals, who may have had more time to take preventive actions, may exhibit lower levels of engagement in implementing further protective measures^31^.

In addition to the regression analyses, we employed LCC models to further explore the segmentation of respondents into distinct groups based on their protection behaviour profiles in response to the Vaia storm. Consistent with the predictions of the PMT theory, our results revealed four distinct classes or groups characterized by different combinations of high and low coping and threat appraisals^34,51^.

The largest group identified, referred to as the “just to be sure” class, exhibited high coping appraisal but did not perceive the threat as severe. Consequently, individuals in this class were less inclined to modify their behaviour in response to the storm. This finding aligns with previous studies that identified similar profiles of individuals who exhibit cautious behaviours without perceiving the threat as significant^34^. The second-largest class, labelled the “problem-focused” group, displayed high threat and coping appraisals. This group primarily consisted of women, a finding consistent with a recent study in the Philippines^52^. Furthermore, our analysis shows that this subgroup predominantly consists of older individuals. This finding accentuates the significance of age as a key factor influencing adaptive responses to storm events. Older individuals within this class, often possessing valuable experiences and a sense of ownership over affected properties, demonstrate a proactive approach to risk mitigation through problem-focused coping strategies. These results align with earlier research conducted on communities in the Eastern Italian Alps that experienced extreme climate events^17^. In that study, three groups of residents were identified based on flood risk awareness and protective behaviours, including a group characterized by low-risk awareness, similar to our “problem-focused” class^17^.

Interestingly, we identified a small class of individuals exhibiting maladaptive responses to the storm. These individuals perceived the threat but lacked trust in current structural flood protection measures or confidence in their ability to carry out protective measures. This group’s vulnerability to fatalistic attitudes and their limited engagement in protective behaviours have also been observed in previous studies^17,51^. The maladaptive behaviour observed in this class specifically involves a hesitancy to rely on existing flood protection infrastructure and a perceived inability to effectively implement personal protective measures. This reluctance may stem from a combination of factors, including a lack of confidence in the efficacy of available measures and a potential sense of disempowerment, which could contribute to heightened exposure to climate risks^53^.

In our study, we aimed to address a gap identified in the literature regarding the relationship between intentions and behaviours in the context of environmental behaviour^54^. Specifically, we examined how non-protective responses, such as denial, fatalism, and wishful thinking^25^, aligned with the profiles identified in our study. Our findings revealed that individuals classified in the maladaptive class tended to maintain their usual behaviour patterns and engaged in daily activities during the event, such as going to work or grocery shopping. On the other hand, the theory predicted that individuals in the “just to be sure” class, characterized by a higher coping appraisal, would have a stronger intention to engage in protective behaviours. Our intention-behaviour check supported this prediction, which showed a higher number of individuals renouncing their routine activities among those belonging to the “just to be sure” class^55^.

The results of our research provide valuable insights into societal responses to extreme events, particularly in understanding why certain groups within communities engage in protective behaviour when faced with catastrophic flood events. These findings contribute to a better understanding of individual risk mitigation decisions, which can inform the development of effective risk management strategies aimed at enhancing citizens’ preparedness and adaptive capacity. Our study confirms the influence of PMT constructs on protective behaviour and identifies other influential factors such as age, gender, previous experience, and residing in high-risk areas. Understanding these factors can inform targeted communication strategies for different groups. For example, our findings support the idea that maintaining high levels of risk perception (threat appraisal) through effective communication can increase the adoption of protective measures in households. This is in line with ref.^56^, who utilized 3D animation to enhance the effectiveness of warning messages in motivating people to take action during flood events. Their study emphasized the importance of combining PMT components, such as highlighting the low cost of taking action, the efficiency of recommended actions, self-efficacy, and the severity of the threat, in communication strategies.

In conclusion, our study highlights the importance of human behaviours and behavioural change in addressing climate change and its impacts. Previous experiences and trust in authorities were found to influence household protective behaviours. While information and warnings had limited reach, they showed potential to increase adaptive coping strategies. Policy efforts should prioritize providing information, especially in high-risk areas, and target tenants and individuals with low protection motivation and coping capacity. Decision-makers should pay special attention to individuals in the “maladaptive” class to reduce maladaptation. Our findings show that by increasing coping appraisals and discouraging non-protective measures, we can work towards reducing maladaptation and promoting effective adaptation strategies.

### Supplementary Information

Supplementary Information.

## Data Availability

The datasets used and analyzed during the current study are available from the corresponding author upon reasonable request.
