# Supplementary material for: Identification of maladaptive behavioural patterns in response to extreme weather events
Source: Sci Rep. 2024 May 8;14:10563. doi: 10.1038/s41598-024-60632-3 (PMC11078959; doi:10.1038/s41598-024-60632-3)
Supplement: Supplementary file 1 — Supplementary Information. [file 41598_2024_60632_MOESM1_ESM.docx]

**Title: Identification of maladaptive behavioural patterns in response to extreme weather events**

**Authors**

Luisa Eusse-Villa^1^ (luisafernanda.eussevilla@phd.unipd.it), Carolina Bonardi Pellizzari^1^ (carolina.bonardipellizzari@unipd.it), Cristiano Franceschinis^1^ (cristiano.franceschinis@unipd.it), Mara Thiene^1^ (mara.thiene@unipd.it), Marco Borga^1^ (marco.borga@unipd.it), and Anna Scolobig^2,3^ (anna.scolobig@unige.ch).

**Affiliations**

^1^ Land, Environment, Agriculture and Forestry Department, University of Padova, Legnaro, Italy.

^2^ Environmental Governance and Territorial Development Institute, University of Geneva, Geneva, Switzerland.

^3^ Equity and Justice Group, International Institute for Applied Systems Analysis, Laxenburg, Austria.

**Corresponding author**

Luisa F. Eusse-Villa ([luisafernanda.eussevilla@phd.unipd.it](mailto:luisafernanda.eussevilla@phd.unipd.it))

**Author contributions**

M.T., M.B. and A.S conceived and designed the study. C.F., L.E.V and C.B.P contributed to data analysis and interpretation. L.E.V and C.B.P. drafted the manuscript. C.F., M.B., A.S. and M.T. reviewed the manuscript.

**Competing interest declaration**

The authors declare no competing interests.

**Availability of Data and Materials**

The datasets used and analysed during the current study are available from the corresponding author upon reasonable request.

**Supplementary Material**

**Supplementary Table 1 |** Behaviour of interest

| **Item** | **Type of variable** | **Question** |
| --- | --- | --- |
| Protective measures before Vaia | Dummy. At least one measure (1), no measure (0) | Barriers to doors and windows on the lower floors of the house |
|  |  | Drainage channels |
|  |  | Pumps |
|  |  | Tree pruning within the property |
|  |  | Sensitive rooms (e.g., bedroom) located on the superior floors |
|  |  | Water removal |
|  |  | Removal of debris / mud |
|  |  | Securing of objects at risk |
|  |  | Sandbags |
|  |  | First aid kit |
|  |  | Flood damage insurance |
|  |  | Aid to people in difficulty |
|  |  | Construction of temporary barriers |
| Protective measures after Vaia | Dummy. At least one measure (1), no measure (0) | Barriers to doors and windows on the lower floors of the house |
|  |  | Drainage channels |
|  |  | Pumps |
|  |  | Tree pruning within the property |
|  |  | Sensitive rooms (e.g., bedroom) located on the superior floors |
|  |  | Sandbags |
|  |  | First aid kit |
|  |  | Flood damage insurance |
| Renounced activities | Dummy. Renounced at least one activity (1), did not renounce any activity (0) | Staying home |
|  |  | Going to work |
|  |  | Going to school/university |
|  |  | Grocery shopping |
|  |  | Accompany/pick up children from school |
|  |  | Visit relatives/friends |
|  |  | Playing sports |
|  |  | Practicing outdoor hobbies |
|  |  | Doing volunteering activities |

**Supplementary Table 2 |** Variables used in the OLS regression and LCC models as explanatory.

| **Item** | **Component** | **Type of variable** | **Variable** | **Question** |
| --- | --- | --- | --- | --- |
| Threat Appraisal | Perceived vulnerability | Continuous variable. Mean score of three statements | TA1 | Events like VAIA have become a serious threat to humanity |
|  |  |  | TA2 | Extreme weather events will be more and more frequent in the future |
|  |  |  | TA3 | I am vulnerable to the negative effects of events like VAIA |
|  | Perceived severity | Continuous variable. Mean score of four statements | TA4 | Another event like VAIA will have long-term negative consequences on the area where I live |
|  |  |  | TA5 | If an event such as VAIA occurs again in the future, my home will be affected |
|  |  |  | TA6 | If another phenomenon such as VAIA recurs in the future, it will be difficult for me to carry out normal activities for the duration of the event |
|  |  |  | TA7 | The negative impacts of events such as VAIA are severe |
|  | Fear | Continuous variable. Mean score of two statements | TA8 | The thought of VAIA-like events scares me |
|  |  |  | TA9 | After VAIA I am afraid to leave my home on bad weather days |
| Coping Appraisal | Response efficacy | Continuous variable. Mean score of six statements | CA1 | The protection against natural disasters is the responsibility of the state, not of the citizens |
|  |  |  | CA2 | The presence of embankment limits the risk of flooding |
|  |  |  | CA3 | The presence of firefighters and civil protection in the area reduces the risks for the population. |
|  |  |  | CA4 | The presence of warning systems makes it possible to reduce damage in situations that are risky for the population |
|  |  |  | CA5 | Personal protection measures are effective in preventing any dangers related to events similar to VAIA |
|  |  |  | CA6 | Insurance policies are a useful tool for economic protection against possible damage |
|  | Self-efficacy | Continuous variable. Mean score of three statements | CA7 | I am not worried about the possible difficulties I might run into due to an extreme event, because I have faith in my abilities to deal with them |
|  |  |  | CA8 | I feel I have control over what happens in emergency situations |
|  |  |  | CA9 | I consider myself a competent person in adopting protective and preventive measures |
|  | Response cost | Continuous variable. Mean score of three statements | CA10 | Risk prevention measures have too high a cost compared to the real benefits they bring |
|  |  |  | CA11 | Taking preventive and protective measures requires me too much time |
|  |  |  | CA12 | Citizens should contribute financially, in addition to the taxes they already pay, so that the State, the Region and the Province implement risk mitigation measures |
| Source of information | Previous experience | Dummy. Yes (1), No (0) | SI1 | Did you experience a similar event before |
|  | Information | Dummy. Yes (1), No (0) | SI2 | Did you receive any information about the danger of the event before or during it |
|  | Source of information | Dummy. Official (1), Otherwise (0) | SI3 | From which source you received the information (official sources, journalists, meteorological service, friends and relatives, others) |
|  | Volunteer activities | Dummy. Yes (1), No (0) | SI5 | Do you volunteer in emergency response organisations |
|  | Personality traits | Continuous variable. Mean score of five statements | SI6 | The warnings and information received worried me |
|  |  |  |  | The warnings and information were relevant to me |
|  |  |  |  | I have taken the warnings and information received seriously |
|  |  |  |  | In retrospect, I found the warnings and information to be exaggerated |
|  |  |  |  | In retrospect, I found the warnings and information insufficient |
|  | Risk zone | Dummy. Yes (1), No (0) | SI7 | Do you live in a risk area (landslide, flood, avalanche) |
| Sociodemographic characteristics | Gender | Dummy. Female (1), Male (0) | SD1 | Gender |
|  | Age | Continuous variable | SD2 | Year of birth |
|  | Education | Dummy. University diploma (1), otherwise (0) | SD3 | Education level |
|  | Income | Dummy. Above 30,000 €/year (1), below 30,000 €/year (0) | SD4 | Annual household income after taxes (€) |
|  | Ownership | Dummy. Own house (1), rented house (0) | SD5 | Is the house you live in yours or do you rent it |

**Supplementary Table 3 |** Criteria for the PMT latent class models

| **Number of classes** | **Model without covariates** | | |  | **Model with covariates** | | |
| --- | --- | --- | --- | --- | --- | --- | --- |
|  | **AIC** | **BIC** | **AIC_c_** |  | **AIC** | **BIC** | **AIC_c_** |
| 2 | 77063.29 | 77948.11 | 76340.10 |  | 76923.9 | 77866.31 | 76149.91 |
| 3 | 75857.89 | 77187.74 | 74727.57 |  | 75648.64 | 77093.67 | 74410.01 |
| **4** | **74613.83** | **76388.70** | **73131.44** |  | **74550.73** | **76498.38** | **72797.05** |
| 5 | 73508.82 | 75728.72 | 71421.77 |  | 73338.62 | 75788.89 | 70949.44 |
| 6 | 72800.16 | 75465.09 | 70172.84 |  | 73348.31 | 75798.58 | 70998.64 |
| 7 | 72540.92 | 75650.88 | 69273.55 |  | 72138.16 | 75593.67 | 68297.99 |

Note: the bolded line highlights the most appropriate model for the purpose of our study


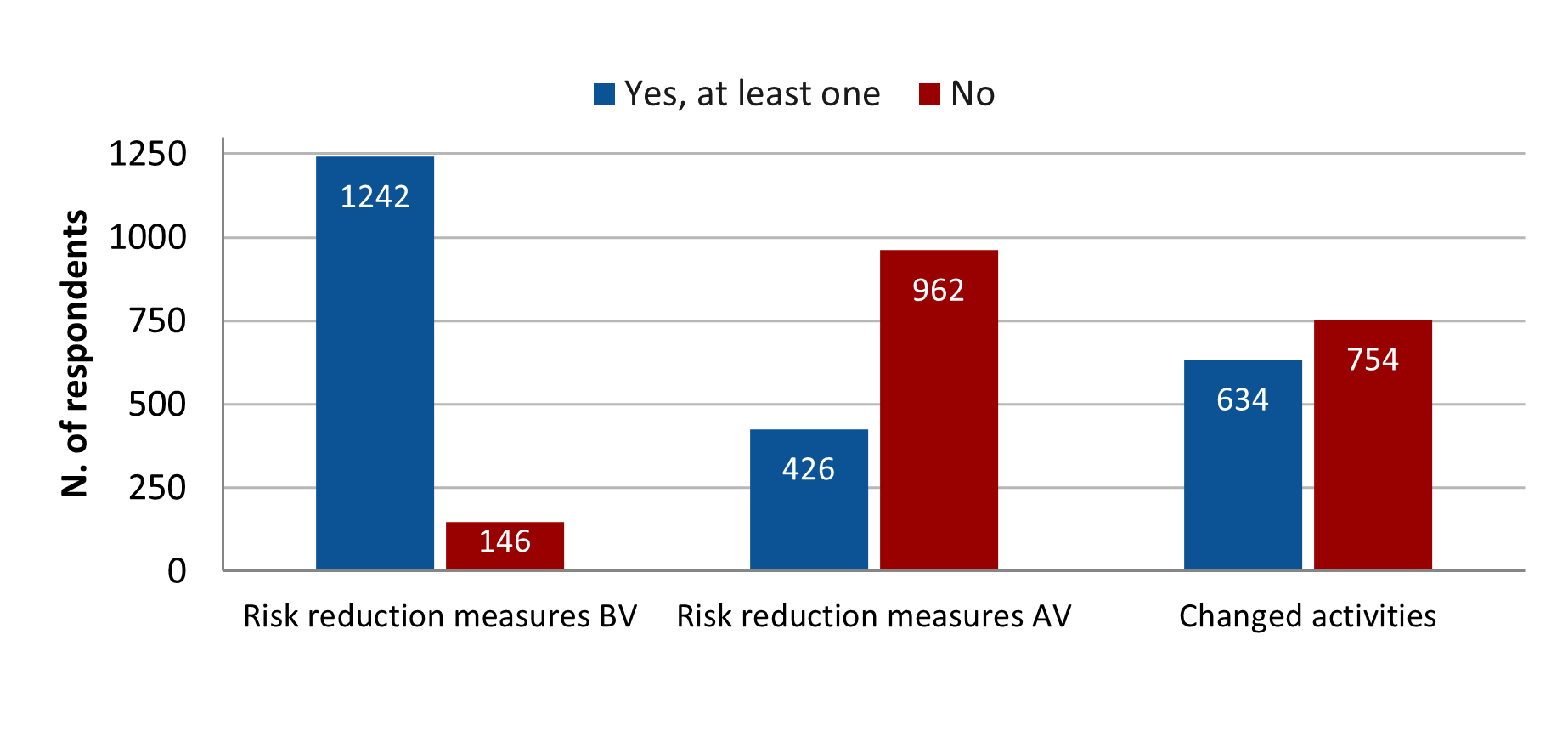


**Supplementary Fig. 1** | **Distribution of respondents who took at least one protective measure and renounced at least to one activity due to the storm.** The blue colour indicates the respondents who engaged in protective behaviour (before and after the storm), while the red colour indicates those who did not take any protective measure or did not renounce to any activity. For details on the protective measures and renounced activities, refer to Supplementary Table 2. Note BV = Before Vaia, AV = After Vaia.
